# War Exposure and Canine Cortisol Responses: Country Differences in Cortisol Profiles of Therapy Dogs

**DOI:** 10.3390/ani16030381

**Published:** 2026-01-25

**Authors:** Sandra Foltin, Svitlana Kostenko, Ann-Danielle Hartwig, Lisa Maria Glenk

**Affiliations:** 1 Department of Biology, University of Duisburg-Essen, 45141 Essen, Germany; 2 Department of Animal Genetics, Breeding and Biotechnology, National University of Life and Environmental Sciences of Ukraine, 15 Heroiv Oborony Str., 03041 Kyiv, Ukraine; 3 Department of Psychology, Witten/Herdecke University, Alfred-Herrhausen-Str. 50, 58448 Witten, Germany; 4 Karl Landsteiner Research Institute for Neurochemistry, Neuropharmacology, Neurorehabilitation and Pain Treatment Mauer-Amstetten, 3362 Mauer-Amstetten, Austria

**Keywords:** therapy dog, war, PTSD, mental health, Ukraine, dog-assisted intervention, cortisol, welfare, stress, dog-assisted services

## Abstract

Dog-assisted interventions (DAIs) are an established procedure to support military staff, but their implementation during active warfare has not yet been systematically studied. In addition, the welfare of therapy dogs participating in DAIs during war remains unexplored. Therapy dogs may develop clinically relevant emotional disorders, including trauma-related stress responses, analogous to human psychopathologies. The present study sought to monitor physiological arousal in therapy dogs performing DAI sessions with their handlers in two Ukrainian military hospitals (Vinnyzja and Kyiv). Biomarkers of hypothalamic–pituitary–adrenal (HPA) axis activity, including salivary, urinary, and hair cortisol concentrations, were assessed in Ukrainian (UA) therapy dogs to capture their acute and long-term stress responses. Additionally, cortisol levels in German (GE) therapy dogs performing similar DAIs under peaceful conditions were measured to compare cortisol levels between dogs from both regions. Results suggest that GE therapy dogs exhibited significantly higher urinary concentrations, reflecting alterations in longer-term glucocorticoid secretion that is possibly caused by war-related stimulation in the UA cohort. In contrast, no significant differences in salivary cortisol emerged as a consequence of performing DAIs. The present findings suggest that the environment rather than involvement in DAIs has an impact on therapy dogs’ cortisol secretion.

## Simple Summary

Dog-assisted interventions (DAIs) are utilized in numerous settings, including treating post-traumatic stress disorder in military personnel. However, implementation during active warfare and the welfare of the dogs involved have not been measured yet. This study evaluated stress-related hypothalamic–pituitary–adrenal (HPA) axis processes in therapy dogs involved in DAIs in Ukraine and in a control cohort in Germany. Salivary, urine, and hair cortisol concentrations were measured to assess acute and long-term stress. Ukrainian dogs displayed significantly lower urinary cortisol levels compared to German dogs, suggesting altered long-term glucocorticoid secretion associated with environmental stressors related to war. No significant salivary cortisol changes were observed in relation to DAI sessions. These findings indicate that environmental conditions, rather than participation in DAIs, may influence stress physiology in therapy dogs.

## 1. Introduction

On 24 February 2022, the Russian Federation launched a full-scale invasion of Ukraine, initiating a prolonged armed conflict with profound humanitarian consequences. War and large-scale crises constitute complex, multidimensional challenges, producing enduring physical, psychological and emotional damages affecting not only human but also non-human animals [1,2,3] working and living in affected environments [3,4,5,6]. From a One Health perspective, the tripartite system involving the environment, humans, and non-human animals is deeply interlinked and essential when assessing mental health outcomes and welfare risks [7,8].

A substantial body of research examined the mental health outcome of war exposure in civilians, refugees, and military personnel. The most prevalent disorders, described post-deployment or post-conflict, are post-traumatic stress disorder (PTSD), depression, and anxiety [9,10,11,12]. Exposure to traumatic events involving threats to life or physical integrity markedly increase mental health disorders, with PTSD being the most prominent and debilitating [2,10,11]. Given its severity, chronicity, and impact on functioning, PTSD warrants special attention in comparative health research on both human and non-human animals. Empirical evidence addressing psychological stress during active warfare, particularly referring to animals, has so far remained scarce [13,14,15].

Studies manifest that dogs are capable of developing clinically relevant behavioral, emotional, and physiological disorders analogous to human psychopathologies, including trauma-related stress responses [3,5,6]. Chronic stress exposure, cumulative trauma, social instability, and insufficient recovery periods—hallmarks of war environments—are known contributors to anxiety disorders, impaired social functioning, and avoidance behaviors in dogs [3,5,6]. Salden et al. (2023) [16], in their systematic review on the behavioral, emotional, and psychological effects of exposure to high-stress and/or traumatic environments, described increased anxiety, hypervigilance, and PTSD in affected dogs. War impacts humans and dogs alike; all experience and endure the invasion accompanying numerous stressors, such as noise, loss, stress, disruption to life, grief, and pain [5,6,17], creating deeply intertwined histories of trauma and adaptation strategies [2,17,18].

Animal suffering in war does not invariably derive from deliberate harmful human treatment; the worst outcomes frequently result from the structural violence inherent in the organized activity that represents war as such. Both endure trying circumstances with challenging travel, life, and logistics [19,20]. The exigencies of war, however, produce the most egregious examples of abuse, with thousands of dogs, horses, or zoo animals involuntarily exposed to suffering and death, including mass execution [18,19,20]. Shorn of unexamined anthropocentric suppositions, the narrative of non-human animals in war unveils various facets of the nature of human relationships with other animals. However, we also realize that the human–dog partnership, derived from the needs and vulnerabilities shared, testifies to human dependence for this extra-species comfort [18]. The roles dogs had and have in war are wide-ranging: they are mine detectors, trench guards, food bearers, and message or equipment carriers underground and across no-man’s land [20,21]. Patron, a terrier trained as an explosive detection dog, is a symbol and mascot of Ukrainian resilience. The dog was awarded a medal for dedicated services by president Selenskyj in May 2022 [22]. However, dogs also serve as emotional support, confidants, and comrades, which illustrates both their instrumental value and exposure to harm [23]. Kateryna et al. (2023) demonstrated that companion animals significantly support civilians’ emotional regulation, reducing anxiety, feelings of helplessness, abandonment, and fear during air raids and displacement [24]. Dogs provide constant companionship and physical closeness, routine, and a sense of responsibility, helping individuals preserve psychological stability amid chaos. These findings align with broader evidence that dogs promote emotional regulation through non-judgmental presence, tactile comfort, and reinforcement of safety and hope [24,25]. The bidirectional bond likely enhances resilience for both humans and dogs, although it might expose dogs to secondary or synchronized stress [26].

Within the military context, the dog–human bond is particularly pronounced. Trust, reliance, and interdependence between soldier and dog have historically enhanced operational effectiveness and psychological resilience [20,27,28]. Rethinking the roles of dogs in war brought about novel ideas and concepts: In the U.S., programs were developed in the early 2000 in which veterans with PTSD were being treated by pairing them with dog–human teams for goal-orientated dog-assisted interventions (DAIs) [27,28,29]. Widespread evidence supports the effectiveness of such programs [27,28]; nonetheless, the implementation of DAIs during active armed conflict and its implication for canine stress, welfare, and ethical responsibility has, to date, not been systematically examined.

From a One Health and welfare perspective, it is critical to recognize that canine wellbeing is closely linked to the animal handler’s mental health [26,29,30]. Psychological distress in handlers has been shown to correlate with increased stress-related behaviors in their dogs, underscoring the bidirectional nature of human–animal emotional transmission [26,30,31] and reflecting psychological and physiological spillover within human–dog teams. This interdependence raises ethical concerns where both human and dog participants are exposed to heightened and persistent stressors [4,26,30]. Therefore, evaluating stress responses in therapy dogs working in wartime environments is essential not only for safeguarding animal welfare but also for ensuring the sustainability and ethical integrity of DAIs. Addressing this gap will contribute to a more comprehensive understanding of shared vulnerability, resilience, and guidance across species in armed conflict.

Stress is an adaptive psycho-physiological response to actual or perceived threats, enabling an organism to efficiently cope with challenges. Initially, noradrenaline is secreted from the locus coeruleus in the brain stem to mobilize the body by facilitating actions that promote survival [17,32]. Additionally, the hypothalamic–pituitary–adrenal (HPA) axis is stimulated to release glucocorticoids to modulate metabolic rates. Behavioral consequences of these adaptations include heightened arousal and vigilance. Under acute conditions, these dynamics typically subside once the stressor is removed [32]. However, when stress persists over time in high intensity, or is poorly regulated, it can lead to chronic dysfunction of the HPA axis, causing prolonged elevations in cortisol which, in turn, precede adrenal exhaustion once bodily resources are depleted [32]. Elevated stress levels have been identified as a key psychological consequence in humans during the ongoing Russian invasion of Ukraine [17,33,34].

Stress reactions to traumatic events can be acute or chronic. Acute stress involves intense emotional and physiological responses that occur within the first month following trauma exposure and gradually diminish [35]. For some individuals, however, distress persists or intensifies over time. Prolonged or repeated exposure to stressors such as violence, displacement, or bereavement substantially increase the threat of developing chronic psychological conditions, including PTSD [35,36]. Although these dynamics are well documented, little attention has been paid to dogs exposed to such stressors.

While most existing studies have examined the physiological and emotional states of therapy dogs in health care or education contexts under peaceful conditions [27,28], the present investigation analyzed data gathered during an enduring war situation. We sought to evaluate biomarkers of stress-related arousal in Ukrainian (UA) therapy dogs who teamed up with their handlers to visit PTSD-affected military personnel in Ukraine. Assessing complementary matrices of cortisol allows the assessment of both acute (saliva) and integrated/short-term (urine) HPA axis activation, whereas hair cortisol provides a longer-term index of chronic stress [37,38,39]. To perform systematic monitoring of the HPA axis, we collected first-morning urine samples to calculate the urinary cortisol (UCC) and cortisol-to-creatinine ratio (UCCR). Furthermore, we sampled saliva to measure salivary cortisol before and after DAIs. In addition, we analyzed hair samples from all participating dogs. German (GE) therapy dogs performing analogous DAI sessions under peaceful conditions were used as a control cohort.

The primary research questions were as follows: Do dogs working in DAIs with their handlers visiting PTSD-affected soldiers in a wartime setting show evidence of elevated physiological stress? How do acute (saliva), integrated (UCC), and long-term (hair) HPA-related stress measures compare with data gathered from a German cohort of therapy dogs? Secondary aims were to (a) describe inter-individual variability between dogs, (b) examine whether repeated saliva sampling reveals acute stress peaks that are not captured by morning UCC, and (c) discuss welfare implications for sustained deployment in conflict zones.

## 2. Materials and Methods

To demonstrate the effects of DAIs on glucocorticoid secretion in saliva, urine, and hair in dogs from distinct living contexts, namely those in war-affected regions (Kyiv and Winnyzja, Ukraine) and dogs in a non-war environment (Germany), we employed a mixed design with both between-subject factors (Ukraine vs. Germany) and within-subject factors (pre- and post-intervention cortisol levels). To determine cortisol parameters, an enzyme-linked immunosorbent assay (ELISA) was used (LABOklin, Bad Kissingen, Germany).

### 2.1. Therapy Dog Sample

Both in person and online participant recruitment of human–dog teams was carried out in 2025 from January to June. Study respondents were invited by the authors via convenience sampling methodology based on personal invitation or online recruitment on social media. Inclusion criteria for UA and GE therapy dog handlers were adults (18 years or older) and individuals with a valid DAI certificate either awarded by an International Society for Animal Assisted Therapy (ISAAT) or European Society for Animal Assisted Therapy (ESAAT) [40,41]-recognized organization or currently attending an ISAAT- or ESAAT-certified DAI education program. German therapy dogs were dogs living and working with their handlers for at least 6 months in Germany, and Ukrainian therapy dogs were dogs living and working with their handlers for at least 6 months in the Ukraine. Therapy dogs’ characteristics, such as age, sex, and location, are displayed in Table 1 and Table 2.

### 2.2. Dog-Assisted Intervention (DAI)

Each dog was given a standardized acclimatization period of 20 min to freely explore the setting without the recipient or leash or handler intervention to minimize stress related to novelty or transportation. The study protocol consisted of three distinct phases for all sessions, which lasted for approximately 20 min in total.

(1) Introductory phase (approximately 5 min): An introductory phase to familiarize the DAI team and recipient, which involves mostly gentle interactions of mild intensity (i.e., dog exploring, approaching, sniffing, or taking a treat). The therapy dog handler introduces the team and preferred activities of the dog.
(2) Main phase (approximately 10 min): The recipient and DAI team engage in either moderately activating or calming activities involving physical contact with the recipient, playful interactions, or simple observation.
(3) Final phase (approximately 5 min): The therapy dog handler announces the upcoming end of the session, providing positive feedback to the recipient and thanks them for their compliance. Both the therapy dog and recipient are given the opportunity for a final interaction of mild intensity.

### 2.3. Salivary Cortisol Analysis

Cortisol concentrations in canine saliva were determined on a working day with a scheduled DAI session. The pre-intervention sample was gathered after an acclimatization period of 20 min, and the post-intervention sample was collected immediately following completion of the DAI session in the same environment. Thus, saliva samples were taken by the experimenter prior to interaction with recipients and immediately after the interaction using LABOklin saliva swabs (LABOklin, Bad Kissingen Germany). To minimize contamination, food was withheld from each dog for 20 min before saliva collection. To absorb saliva, the swab was gently inserted into the dog’s buccal cavity, targeting the lower gum line behind the last molar, where saliva tends to pool, and held in place for 30 s before being transferred to a collection tube. To estimate baseline variability, each dog was considered its own control. To determine salivary cortisol, an enzyme-linked immunosorbent assay (ELISA) was used (LABOklin, Germany). Salivary cortisol is reported in µg·dL^−1^.

### 2.4. Urine Cortisol Analysis

First naturally voided morning urine samples were collected by the handlers for cortisol analysis to minimize diurnal variation caused by DAI sessions. Urine collection occurred prior to any intervention-related activity and before travel to or arrival at the DAI location. Samples were obtained by using a sterilized ladle, and the urine was immediately transferred using a disposable pipette into a polypropylene tube. Minimum required volume was 0.5–2 mL urine. Samples were handled with minimum delay and stored at a temperature of −20 °C or lower in a freezer. To determine urinary cortisol, an enzyme-linked immunosorbent assay (ELISA) was used (LABOklin, Germany). Urinary cortisol is reported in nmol·L^−1^.

### 2.5. Urine–Cortisol–Creatinine Ratio Analysis

To determine the Urine–Cortisol–Creatinine Ratio (UCCR), the collected morning urine was utilized and processed according to the abovementioned protocol. To measure urinary creatinine, a standard enzymatic assay was used to account for urine concentration variability (LABOklin, Germany). The UCCR corrects for urine concentration, making it more reliable than raw cortisol measurements alone by reflecting integrated cortisol secretion over time. UCCR measures cortisol (and its metabolites) concentrations in urine normalized to creatinine, a muscle-derived metabolite. Dividing cortisol by creatinine, which is excreted at a stable rate, provides a concentration-independent standardized index. The ratio describes cortisol per unit of creatinine, making it possible to compare cortisol measurements from different samples.

### 2.6. Hair Cortisol Analysis (HCC)

A tuft of 2 cm long hair was collected by the experimenter from each dog for cortisol analysis. Samples were retrieved from the base of the back of the neck of each dog by their handler. Collection of samples was performed by cutting the hair as close to the skin as possible without damaging the skin [39,40,41]. The desired weight of the hair sample was 40 mg or more. Collected hair samples were wrapped in aluminum foil to protect them from sunlight and stored at room temperature in a dark environment. Subsequent extraction of cortisol was performed based on the protocol by Davenport, Tiefenbacher, Lutz, Novak, and Meyer (2006) [42]. Hair cortisol (HCC) is reported in pg·mg^−1^. To control for factors like coat color and season, samples from all groups (Germany, Kyiv, and Vinnyzja) were collected within similar time frames and seasons, ensuring comparable variation [42,43].

### 2.7. Statistics

All statistical analyses were performed using JASP software (version 0.18.3 for Windows; JASP Team, 2024). Graphical representations were also generated in JASP. Statistical significance was set at *p* < 0.05 for all analyses. Data were assessed for normality and homogeneity of variance using the Shapiro–Wilk and Levene tests, respectively. As the assumption of normality was violated, nonparametric tests were applied. Specifically, the Kruskal–Wallis ANOVA was used to compare groups, followed by Dunn’s post hoc test for pairwise comparisons. Protein and creatinine concentrations in urine, as well as the UCCR, were compared across all dog groups. Data are presented as medians, means, and ranges. Group differences in categorical variables were analyzed using Fisher’s exact test, while differences in continuous variables were examined with the Kruskal–Wallis test. Cronbach’s alpha was calculated to assess the internal consistency reliability of the applied scales.

To analyze differences in stress markers (salivary cortisol [SCC], urinary cortisol [UCC], and hair cortisol [HCC]) and UCCR among dog groups at two levels—Level 1: Kyiv (KY) vs. Vinnyzja (VI) vs. Germany (GE), and Level 2: Ukraine (UA) vs. Germany (GE)—one-way ANOVAs were performed with group as the fixed factor and cortisol markers as dependent variables (α = 0.05). In cases of variance heterogeneity or violations of normality assumptions, Welch ANOVAs were applied. For post hoc comparisons in the three-group analysis, Games–Howell tests were conducted.

Effect sizes were calculated as Cohen’s d for *t*-tests and partial eta-squared (η_p_^2^) for ANOVAs. Following established conventions, Cohen’s d values of 0.20, 0.50, and 0.80 represent small, medium, and large effects, respectively [44]. Similarly, η_p_^2^ values of 0.01, 0.06, and 0.14 correspond to small, medium, and large effects, respectively [45,46].

## 3. Results

### 3.1. Demographics

As depicted in Table 1, the final sample consisted of *n* = 56 dogs. On average, dogs had a median age of *Md* = 4.0 years (*IQR* 3.5, range 1–19 years), with 27 female (48.2%) and 28 male dogs (50%). One dog’s sex had not been documented.

In Table 2, the UA therapy dog cohort was divided into subgroups, resulting in k = 3 groups overall, of which 19 therapy dogs were assessed in Kyjiw (KY therapy dogs) and 20 in Vinnyzja (VI therapy dogs). In Germany, 17 therapy dogs participated (GE therapy dogs).

In summary, the sample sizes did not differ significantly (F3 groups (2,53) = 1.96, *p* = 0.15, η^2^ = 0.07; F2 groups (1,54) = 3.83, *p* = 0.06, η^2^ = 0.07). Sex was equally distributed ((χ^2^ 3 groups (2, *n* = 55) = 2.67, *p* = 0.26, V = 0.22; χ^2^ 2 groups (1, *n* = 55) = 0.04, *p* = 0.84, V = 0.03), while the Shapiro–Wilk test for age was significant (W years = 0.84, *p* < 0.001; W months = 0.85, *p* < 0.001).

Using Independent Samples *t*-tests, we examined whether the dog’s sex influenced cortisol markers. No significant differences could be found between male and female dogs in their cortisol levels, neither for their UCC (t(49) = 1.76, *p* = 0.20, d = 0.29) nor for their HCC (t(39) = 1.67, *p* = 0.10, d = 0.32), or the pre and post intervention values of the SCC (t(26) = 0.90, *p* = 0.38, d = 0.34, t(19) = 0.87, *p* = 0.40, d = 0.42).

### 3.2. Salivary Cortisol (SCC)

**Figure 1:**
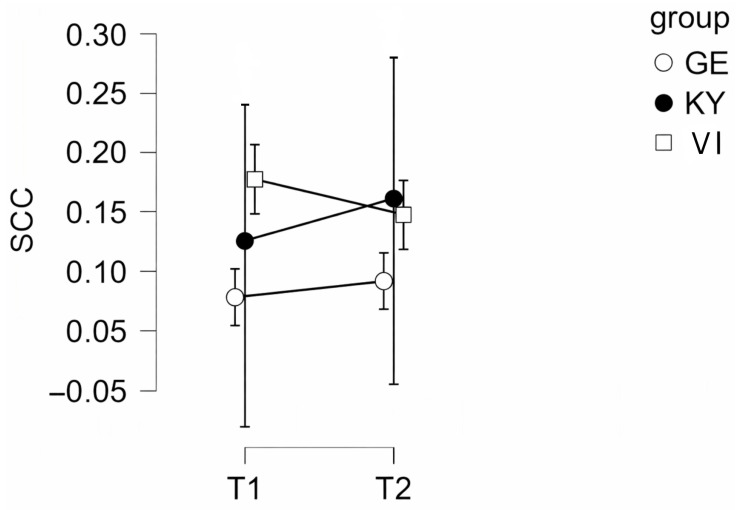
Salivary cortisol concentrations (SCC, expressed in µg/dL) before (T1) and after (T2) a DAI session in UA therapy dogs (KY therapy dogs and VI therapy dogs) and GE therapy dogs. GE, German; UA, Ukrainian; KY, Kyiv; VI, Vinnyzja.

To examine potential group differences, time effects, and their interaction, mixed-design ANOVAs were conducted. All assumptions were tested and met. Sphericity was not a concern because the within-subjects factor included only two measurement points. Homogeneity of error variances was confirmed using Levene’s test. The mixed-design ANOVA comparing the three groups (Kyiv [KY], Vinnyzja [VI], and Germany [GE]) revealed no significant main effect of time (F (1, 12) = 0.23, *p* = 0.64, η_p_^2^ = 0.02), indicating that salivary cortisol levels did not significantly change from before to after the intervention (see Figure 1).

The main effect of group was not statistically significant (F (2, 12) = 3.11, *p* = 0.08, η_p_^2^ = 0.34) but showed a statistical trend toward group differences. No significant time × group interaction was found (*p* > 0.05), suggesting that changes in cortisol levels over time did not differ between groups.

### 3.3. Urinary Cortisol (UCC)

**Figure 2:**
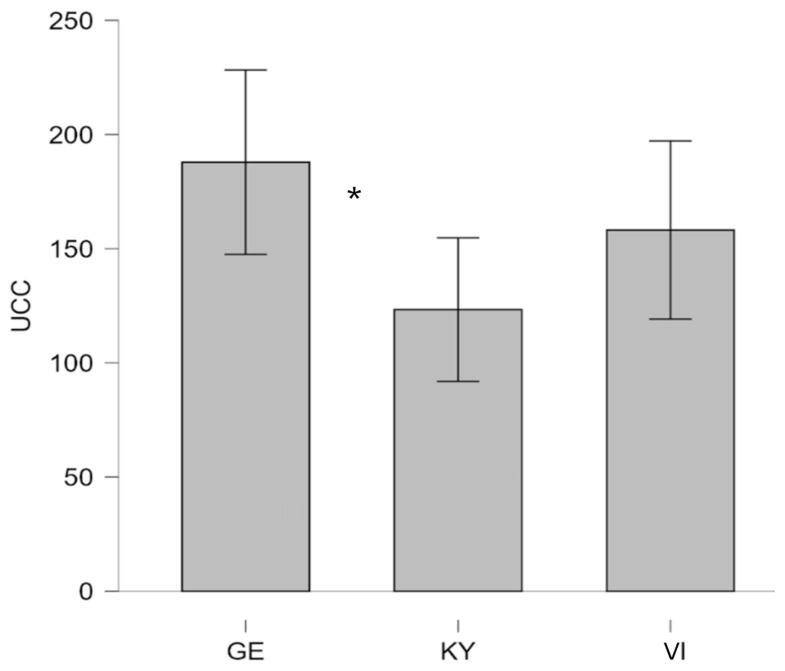
Urinary cortisol concentrations (UCC, expressed in nmol·L^−1^) in UA therapy dogs (KY therapy dogs and VI therapy dogs) and GE therapy dogs. GE, German; UA, Ukrainian; KY, Kyiv; VI, Vinnyzja. An asterisk (*) indicates statistical significance at *p* < 0.05.

For the UCC, homogeneity of variances was asserted using Levene’s test, which showed that equal variances could be assumed for both levels of group comparisons (p3 groups = 0.54; p2 groups = 0.90). As shown in Figure 2, GE therapy dogs had the highest mean UCC levels (M = 187.9, SD = 78.57, *n* = 17), followed by VI therapy dogs (M = 158.2, SD = 75.81, *n* = 18), with KY therapy dogs showing the lowest levels (M = 123.4, SD = 63.15, *n* = 17). Post hoc Tukey comparisons revealed that this effect was driven by a significant difference between the GE and KY groups (MDiff = 64.51, *p* = 0.03, d = 0.89), marked by a large effect size, with GE therapy dogs exhibiting substantially higher UCC levels than those from Kyiv.

**Figure 3:**
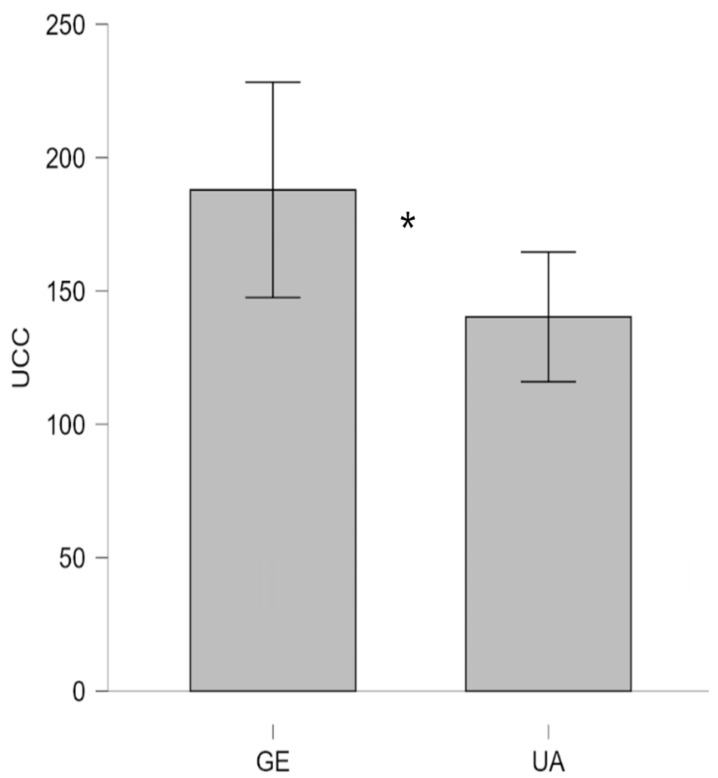
Urinary cortisol concentrations (UCC, expressed in nmol·L^−1^) in UA therapy dogs and GE therapy dogs. GE, German; UA, Ukrainian. An asterisk (*) indicates statistical significance at *p* < 0.05.

When the data were analyzed using a two-group comparison (Ukraine [KY + VI] UA vs. Germany [GE]), the one-way ANOVA also revealed a significant main effect with a medium effect size (F (1, 50) = 4.82, *p* = 0.03, η_p_^2^ = 0.09). As depicted in Figure 3, the data shows that dogs from Germany had significantly higher UCC levels (M = 187.9, SD = 78.57, *n* = 17) compared to dogs from Ukraine (M = 140.3, SD = 70.79, *n* = 35).

### 3.4. Urine–Cortisol–Creatinine Ratio Analysis (UCCR)

UCCR values of the majority of dogs were within the physiologically acceptable reference range (<10–12) but with substantial inter-individual variability (see Table 3). In general, UCCR values ranged from 0.18 to 41.44 with a median of 8.98. Three individuals from the VI cohort displayed very low scores (0.2), and two were extreme outliers with a marked increase (33.46 and 41.44). From the GE cohort, one dog exhibited a very high value (33.14). No extreme values were found in the KY cohort.

### 3.5. Hair Cortisol Concentrations (HCCs)

**Figure 4:**
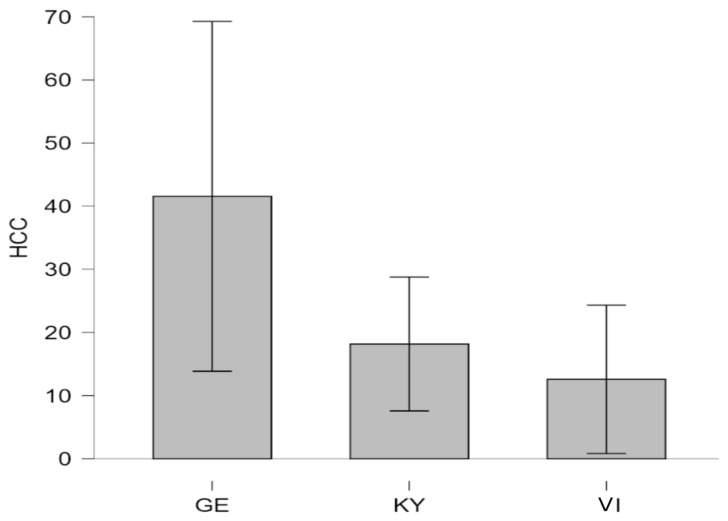
Hair cortisol concentrations (HCCs, expressed in pg·mg^−1^) in UA therapy dogs (KY therapy dogs and VI therapy dogs) and GE therapy dogs. GE, German; UA, Ukrainian; KY, Kyiv; VI, Vinnyzja.

HCC was assessed as a third and more long-term indicator of cortisol levels. Levene’s test revealed that the assumption of equal variances was violated for both levels of group comparisons (three-group comparison: *p* = 0.002; two-group comparison: *p* < 0.001). Consequently, Welch’s ANOVA and Games–Howell post hoc tests were applied. The one-way Welch ANOVA comparing the three groups (Kyiv [KY], Vinnyzja [VI], and Germany [GE]) did not show a significant main effect of location on HCC (F (2, 21.47) = 2.21, *p* = 0.13, η_p_^2^ = 0.11). In the absence of statistical significance, the pattern of group means followed the same direction as observed for UCC levels. GE therapy dogs showed the highest mean HCC (M = 41.56, SD = 52.00, *n* = 16), followed by KY therapy dogs (M = 18.17, SD = 5.04, *n* = 19), and VI therapy dogs had the lowest levels (M = 12.58, SD = 11.18, *n* = 6), as illustrated in Figure 4.

Accounting for the absence of statistical significance, no further comparison between the UA and GE cohorts was made. Of note, a significant correlation was found between HCC and urinary cortisol-to-creatinine ratios (τ = 0.30, *p* < 0.001).

## 4. Discussion

Previous studies have emphasized that dogs participating in therapeutic programs are exposed to both endogenous and exogenous stressors, which may deeply impact their behavior, health, and emotional wellbeing [47,48,49]. Dogs participating in DAI occupy a dual role: they provide psychosocial and physiological benefits to vulnerable humans while themselves being exposed to the emotional and physical demands of the intervention setting [49]. Because animal welfare and handler–dog functioning are crucial for sustainable, ethical DAI deployment, objective physiological measures of stress in dogs are essential. Cortisol, as the primary glucocorticoid produced by the adrenal glands, has vital regulatory effects on physiology and metabolism. Secreted in high levels, it impacts glucose homeostasis, mediates stress adaptation mechanism, and modulates immune functions, thereby modeling the dogs’ comprehensive metabolic state [32,50,51].

### 4.1. Salivary Cortisol (SCC)

To better understand the impact of DAI on canine welfare, salivary cortisol was measured in participating dogs as a physiological indicator of stress. Cortisol, a key hormone of the HPA axis, is central in regulating emotional arousal, energy mobilization, and adaptive responses to environmental demands [32,50,52]. As a non-invasive measure, it offers insight into neuroendocrine pathways, reflecting acute physiological and emotion states [53,54,55]. In our study, SCC levels did not change significantly pre- to post-intervention. This suggests that the activities involved did not elicit acute physiological stress responses in the participating dogs. Likewise, no significant interaction between time and group was observed, demonstrating comparable cortisol stability across the different locations. Although the main effect of the group was not statistically significant, the pattern of means indicated slightly higher SCC concentrations in GE therapy dogs compared to UA therapy dogs, suggesting potential contextual or environmental influences on baseline stress levels rather than intervention-related effects. Of note, a bigger sample size or an altered statistical threshold (α = 0.10) could likely lead to statistical significance in SCC. The standard deviation seen in KY therapy dogs could be caused by highly individualized responses to environmental circumstances related to the war as Kyiv remains a strategic Russian target.

Our results are consistent with previous studies reporting that animal-assisted interventions (AAIs) and structured human–dog interactions do not typically elevate cortisol concentrations in well-adapted dogs [48,50,51,56]. Glenk et al. (2014) [53] similarly found that therapy dogs showed stable or even reduced salivary cortisol during animal-assisted sessions, suggesting that positive social engagement can buffer physiological stress responses. Other research also emphasizes the role of habituation and handler familiarity in maintaining low stress indicators during such interactions [29,41,53,54,57]. Overall, the absence of significant changes in salivary cortisol in the present study supports the interpretation that the intervention was not perceived as stressful by the dogs. Instead, it aligns with evidence that dogs accustomed to human interaction and structured tasks can remain physiologically stable during therapeutic or social settings.

### 4.2. Urinary Cortisol (UCC)

UCC represents another non-invasive biomarker of physiological stress in dogs that illustrates the activity of the HPA axis. When a dog experiences a physical or psychological stressor, the HPA axis is stimulated, resulting in the secretion of cortisol from the adrenal cortex [50,52,58]. Cortisol then circulates in the bloodstream and is metabolized and excreted in urine, permitting UCC to function as an integrated measure of systemic cortisol output over several hours prior to sampling [37,59,60,61,62,63].

Variations in UCC occur when an animal’s arousal or stress state shifts—for instance, during exposure to novel environments, social challenges, handling, or physical exertion. Elevated UCC levels usually specify amplified HPA axis activity, while stable or lower levels suggest habituation, calmness, or positive welfare states [59,63,64,65]. UCC provides a reliable and ethically adequate method for evaluating welfare in dogs [37,50,53], and as a welfare indicator, UCC is valued for capturing physiological responses that are not easily observable solely through behavior. UCC offers evidence of how dogs experience and cope with environmental or social conditions [57,58]. Studies frequently utilize UCC to evaluate whether participation leads to stress reduction, arousal, or stability [59,60,66].

In our study, GE therapy dogs displayed the highest mean UCC, followed by VI and KY therapy dogs. These results suggest cross-location variation in basal stress physiology rather than intervention-related effects given the absence of changes in SCC. Comparable findings have been reported in studies demonstrating that environmental context, training, daily routines, and handling practices can influence cortisol excretion in dogs [56,57,58,67]. Consistent with previous research, the absence of a significant time effect or interaction indicates that the intervention itself did not induce quantifiable stress responses [52,54,55,68]. Instead, the observed group alterations may reflect divergent management conditions, social environments, or levels of habituation to human interaction across study sites.

Although GE therapy dogs showed significantly higher urinary cortisol levels than UA therapy dogs, this difference may reflect contextual rather than interventional factors. Several explanations are plausible. GE therapy dogs may experience more structured routines, enrichment and greater coaching, which can elevate baseline cortisol without indicating distress [49,52,55]. In contrast, dogs from Ukraine, particularly those living amid the ongoing war, may have adapted to chronic environmental challenges in ways that alter their stress physiology [55,56,57]. Prolonged or repeated exposure to unpredictable or threatening conditions can lead to hypoactivation of the HPA axis, resulting in lower basal cortisol concentrations—a phenomenon observed in both human and other animals under sustained adversity [59,60,61,62,69].

Thus, the comparatively lower UCC levels in UA therapy dogs may not automatically be interpreted as indicative of better welfare but rather as a possible sign of physiological adaptation or blunted stress reactivity after prolonged exposure to stressors associated with wartime environments, such as noise, displacement, or reduced predictability. Given that the significant elevation in UCC was observed specifically in the Kyiv dogs, these contextual differences support the interpretation that higher basal cortisol may reflect greater cumulative or chronic stress exposure associated with more intense and sustained wartime conditions. While Kyiv and Vinnytsia were grouped in the discussion when referring to hyporegulation patterns at a broader level, the site-specific findings underscore the importance of local war intensity as a contributing environmental factor.

Conversely, higher UCC values in GE therapy dogs may reflect greater stimulation or heightened arousal in more stable and controllable conditions. Future studies combining physiological, behavioral, and contextual data are essential to disentangle whether these differences reflect adaptive modulation or welfare disparities between populations living under markedly different socio-environmental circumstances.

UCCR is the accepted gold standard for quantifying proteinuria in veterinary medicine [63,64,70,71]. It is utilized to evaluate systemic conditions, making it a reliable parameter for longitudinal monitoring [64]. Furthermore, it is a non-invasive biomarker for assessing stress and diagnosing endocrine disorders in dogs. Creatinine, a small molecule formed as a byproduct of muscle metabolism, is mainly influenced by the amount of muscle mass and correlates positively with body weight. UCCR provides a complementary measure to salivary and urinary protocols, and by normalizing cortisol excretion, the effects of urine dilution are minimized. According to Del Baldo et al. (2022), reference UCCR values for healthy dogs are approximately 3.38 × 10^−6^ (range: 1.11–17.32 × 10^−6^) [63]. UCCR values greater than 30 may indicate hyperadrenocorticism, stress, or non-adrenal illness [64]. Zeugwetter et al. (2010) reported median UCCR values of 9.6 × 10^−6^ (range: 3.9–88.2 × 10^−6^), 2.5 × 10^−6^ (1.0–8.2 × 10^−6^), and 14.7 × 10^−6^ (3.2–401.7 × 10^−6^) in healthy dogs [64]. Since all participating dogs were reported to be healthy according to regular veterinary screening, no medical follow-up was scheduled in the study protocol. Thus, it is possible that the outliers may not be maladaptive responses but instead could still be underlying medical illness.

Our UCCR data illustrate, from a stress physiology standpoint, three distinct cortisol phenotypes. The majority of dogs display adaptive stress response levels within normal distribution reference limits. Dogs with markedly elevated UCCR levels display excessive HPS axis activation, and dogs with profoundly low UCCR levels show a suppressed stress response. These patterns underscore UCCR utility as screening biomarker for stress-related endocrine dysregulation, identifying hyper- and hypo-reactive cortisol phenotype deviations displaying maladaptive stress responses.

### 4.3. Hair Cortisol (HCC)

HCC is a relatively recent method used to assess physiological stress. It has to be taken into account that it varies with season, coat type, sex, age, and growth of hair, as it takes varied periods of time before a new segment of hair appears at the skin surface and can be measured [65,72,73]. Comparable to other freely circulating molecules, cortisol becomes incorporated into growing hair follicles and thereby generates a measurable plasma cortisol level. HCC levels intercorrelate with saliva and serum cortisol and make it a valuable tool for assessing long-term physiological stress. HCC evinces cumulative cortisol secretion over weeks to months, integrating repeated activation of the HPA axis into a stable, retrospective marker of physiological stress [65,66]. On the one hand, elevated HCC values can result from sustained extrinsic stimulation, social activity, or ongoing stress exposure. On the other hand, lower concentrations may be indicative of either low stress load or result from chronic HPA axis downregulation following prolonged adversity [65,67,68]. When discussing the higher HCC observed in the GE dogs, it is important to note that the inclusion of additional environmental (e.g., detailed living conditions questionnaires), behavioral (e.g., handler-reported behavior and activity surveys), or physiological measures (e.g., heart rate variability) would have helped to clarify whether the observed differences contributed to the elevated HCC values.

The analysis of HCC revealed no statistically significant effect of location, although the mean values followed the same pattern observed for urinary cortisol. GE therapy dogs displayed the highest average HCC levels, followed by KY and VI therapy dogs. Similarly to SCC, a bigger sample size or an altered statistical threshold (α = 0.10) could likely lead to statistical significance. The observed tendency toward higher HCC in GE therapy dogs may reflect great exogenous engagement and activity levels or stressful living conditions [66,69,70]. Conversely, UA therapy dogs’ lower HCC might indicate adaptive physiological modulation under chronic contextual strain, such as exposure to unpredictable conditions related to the ongoing war, resulting in a blunted cortisol response over time [52,69,70,71].

At first glance, it might seem counterintuitive that dogs living in a war-affected country would show lower long-term cortisol levels than those in peaceful environments. However, this may reflect the body’s attempt to maintain stability under repeated or uncontrollable stress [72,73,74]. In the case of Ukrainian dogs, living for several years amid war may have led to such chronic adaptation. Continuous exposure to unpredictable noise, displacement, and reduced environmental stability could have altered HPA responsiveness, producing lower basal cortisol concentrations [75]. These findings are consistent with previous studies showing that HCC is sensitive to extrinsic and intrinsic factors but not necessarily a direct measure of negative stress [70,71,76]. Instead, HCC provides a long-term index of allostatic load, offering valuable insight into how dogs physiologically adjust to their living contexts.

Similar mechanisms have been described in dogs engaged in highly stressful DAI settings [74,75,77], where initial hypercortisolism is followed by a “burnout” phase with reduced cortisol output [73,77,78]. Lower HCC in chronically ill dogs, for instance, reflected HPA axis adaptation or dysregulation rather than simply lower stress [76,78].

### 4.4. Handler Related Factors

Mitropoulos et al. (2025) demonstrated that HCC in dogs is not only impacted by dog-level variables like age or sex, but also by handler-related factors [75]. They found that older handlers were associated with higher HCC, suggesting that handler demographics and handler–dog attachment influence long-term cortisol parameters in their dogs. Barcelos et al. (2024) in their review likewise emphasized the bidirectional nature of dog–handler relationships, pointing out that handler stress and mental health challenges can exacerbate canine behavioral problems, thereby creating a negative feedback loop [76,77]. A One Health welfare approach is recommended to address both handler and dog wellbeing simultaneously and with an interdisciplinary collaboration [75,76,77].

With reference to handlers, a vital aspect is the necessity of enhanced ethological knowledge and adequate stress perception. Erichsmeier et al. (2025) examined dog behavior during DAIs in concurrence with handlers’ perceptions of their dogs’ stress [77]. They described that dogs displayed numerous observable stress-related behaviors, indicating physiological or emotional arousal and decreased well-being, which may reflect increased allostatic load. Handler perception was incongruous, as they commonly described their dogs as not or only minimally stressed. Despite substantial behavioral indicators of discomfort, handlers frequently underestimate dogs’ stress, which may result in delay or repress adequate rest or workload adjustment. This highlights that objective behavioral monitoring and targeted handler education is pivotal to canine well-being [77,78,79].

In this context, Cortesi et al. (2025) examined stress and burnout in dogs participating in DAIs, analyzing and contrasting it with the handler evaluation [78]. They observed a discrepancy in that handlers commonly recognized acute stress (avoidance behaviors, reduced cooperation) but were seldom able to identify chronic stress, emotional withdrawal, learned helplessness, or burnout in their dogs [78]. These findings suggest that dogs partaking regularly in DAIs may face health and welfare risks. One solution is enhanced and more effective monitoring of the handlers, assessment of high-risk environments, and optimized management strategies for handlers to protect their dogs, in particular regarding duration, frequency, and recovery time of an intervention [50,79]. Given recent research outcomes, reliance on handler opinion alone holds the distinct potential to underestimate cumulative stress and strongly emphasizes the need for standardized welfare guidelines and substantive training programs for all handlers [77,78,79].

Although handler demographic and dog–handler data were collected, they were not analyzed within the scope of this study and will be addressed comprehensively in a subsequent publication.

### 4.5. Animal Welfare Strategies

Strategies to improve canine welfare, especially during wartime and crisis, may focus on providing environmental and social conditions that support both physical and psychological well-being. Environmental enrichment, including physical and cognitive stimulation, agency, and safe exploration, can promote engagement without causing over-arousal [79,80,81]. Predictable routines and safe spaces are essential, and handling schedules can help reduce physiological stress. The quality of the human–dog relationship plays a key role, as caregiver training that encourages positive interactions and minimizes aversive handling can buffer stress responses and support healthy cortisol regulation [76,81,82]. Studies have shown that therapy dogs with handlers who manage stress effectively display lower cortisol reactivity in challenging settings [81,82], supporting the idea that improving handler well-being can directly enhance animal welfare in applied contexts. For instance, Roth et al. (2021) examined cortisol levels in dogs before and after owner-targeted stress reduction programs, reporting modest decreases in canine HCC following improvements in owner well-being [44]. Longitudinal studies incorporating genetic markers, wearable stress monitors, and environmental assessments are expected to further clarify how shared environments and relationships influence this synchronization.

Recognizing this bidirectional relationship has significant implications for both animal welfare and human mental health [44,81,82,83]. Interventions aimed at reducing handler stress may indirectly benefit the dog. Mindfulness, non-aversive positive reinforcement-based training, structured exercise routines, and rituals have been associated with improved behavioral outcomes and reduced stress markers [81,82,83]. Additionally, programs that support owners—such as education on canine body language, consistent routines, and the promotion of secure attachment behaviors—may help to minimize stress transmission within the dyad.

### 4.6. Limitations and Future Directions

Limitations of this study include its cross-sectional design, which prevents causal inferences and potential biases arising from convenience sampling in both Ukrainian and German respondents. Given the exploratory nature of the study, the want of sample representativeness and limited generalizability should be acknowledged. Moreover, the small number of human–dog teams involved further constrains the applicability of the findings to broader populations. Nonetheless, the present results contribute valuable insights into glucocorticoid responses of therapy dogs in DAIs during crisis and warfare. To enhance generalizability, future studies should apply probability sampling methods. Furthermore, longitudinal research designs are recommended to examine recovery trajectories, investigate attachment dynamics between handlers and dogs in conflict settings, and evaluate whether structured interventions can mitigate the psychological and physiological burdens associated with wartime deployment over time. Future studies conducted in war or other crisis settings would benefit from incorporating qualitative methodologies, such as focus groups and multimodal observational approaches, to complement physiological measures and provide a more comprehensive understanding of the lived experiences of therapy dog–handler teams in complex, high-stress environments.

This study provides novel data on stress parameters in therapy dogs operating under active war conditions, a context that has been largely absent from existing research. By demonstrating the relevance of cortisol-based assessment on highly unstable and high-risk environments, this study extends the methodological and informational boundaries of animal-assisted intervention research. These findings contribute to a deeper understanding of how extreme, prolonged stressors affect therapy teams and offer a foundation for developing guidelines to support their welfare in war and other crisis settings.

## 5. Conclusions

Existing research has not yet yielded conclusive evidence regarding the explanatory pathways or the specific physiological mechanisms through which benefits of human–animal interactions are conferred. A more comprehensive synthesis of the available evidence is therefore essential to understand the potential impacts of human–dog interactions on both physical and psychological health, as well as to identify the underlying mechanisms that mediate these effects. Such insights are critical for developing evidence-based approaches to enhance a One Health Welfare approach.

Future research should combine behavioral, physiological, and environmental assessments to predict health outcomes and evaluate how interventions, such as enrichment and structured routines, modulate welfare over time. Longitudinal studies tracking dogs in high-stress contexts would help clarify whether observed cortisol patterns reflect adaptive physiological modulation or indicate hidden welfare risks.

## Figures and Tables

**Table 1:** Age and sex of UA therapy dogs and GE therapy dogs. (GE (German), UA (Ukrainian)).

	Age in Years	
	GE	UA
n	17	39
*Md*	4.0	5.0
*IQR*	3.0	4.0
*W* (Shapiro-Wilk)	0.862	0.856
*p* of Shapiro-Wilk	0.017	<0.001
Minimum	1.000	1.000
Maximum	11.00	19.00
sex: female	*n* = 8	*n* = 19
sex: male	*n* = 9	*n* = 19
sex: missing		*n* = 1

**Table 2:** Age and sex of UA therapy dogs (KY therapy dogs and WI therapy dogs) and GE therapy dogs. (GE (German), UA (Ukrainian; KY = Kyiv, VI = Vinnyzja).

	Age in Years		
	GE	KY	VI
n	17	19	20
*Md*	4.0	5.0	4.5
*IQR*	3.0	4.5	4.25
*W* (Shapiro-Wilk)	0.862	0.895	0.808
*p* of Shapiro-Wilk	0.017	0.040	0.001
Minimum	1.000	1.000	2.000
Maximum	11.00	17.00	19.00
sex: female	*n* = 8	*n* = 7	*n* = 12
sex: male	*n* = 9	*n* = 12	*n* = 7
sex: missing			*n* = 1

**Table 3:** Urine-Cortisol-Creatinine Ratio analysis (UCCR) in UA (KY therapy dogs and WI therapy dogs) and GE therapy dogs. (GE (German), UA (Ukrainian; KY = Kyiv, VI = Vinnyzja).

	GE	KY	VI
UCCR			
*M*	10.78	9.64	10.64
*SD*	6.5	4.32	10.01
*Minimum*	4.63	2.14	0.18
*Maximum*	33.14	17.7	41.44

## Data Availability

Personal data is unavailable due to privacy or ethical restrictions.
